# Blastic Plasmacytoid Dendritic Cell Leukemia Successfully Treated by Autologous Hematopoietic Stem Cell Transplantation to a Remission of 48-Month Duration

**DOI:** 10.1155/2013/471628

**Published:** 2013-12-11

**Authors:** Deniz Goren Sahin, Olga Meltem Akay, Hava Üsküdar Teke, Neslihan Andıc, Eren Gunduz, Zafer Gulbas

**Affiliations:** ^1^Department of Hematology, School of Medicine, Eskisehir Osmangazi University, Meselik, Eskisehir, Turkey; ^2^Department of Hematology, Anadolu Medical Center, Istanbul, Turkey

## Abstract

Blastic plasmacytoid dendritic cell neoplasm (BPDCN) is a very rare subtype of AML characterized by the clonal proliferation of precursors of plasmacytoid dendritic cells. It presents with an aggressive behavior. The clinical findings include cytopenia, particularly thrombocytopenia. Although it responds well to chemotherapy initially, the relapse is a rule and prognosis is very poor. There is limited data published in the literature, making it very problematic to define the biological and clinical features, hence, the appropriate therapeutic approach. There are various treatment methods such as multiagent chemotherapy based on ALL or AML and/or hematopoietic stem cell transplantation. However, none of them is approved as a standard therapy. From this point of view, we herein report a 20-year-old case at onset of a leukemic form of BPDCN who survived 48 months after autologous hematopoietic stem cell transplantation.

## 1. Introduction

Acute myeloid leukemia (AML) and related precursor neoplasm classification are being constantly updated by World Health Organization. Recently, blastic plasmacytoid dendritic cell neoplasm (BPDCN) is categorized under AML related precursor neoplasm subgroup [1]. This is a very rare subtype of AML characterized by the clonal proliferation of precursors of plasmocytoid dendritic cells. Its incidence is as low as 0.44% of all hematologic malignancies and the leukemic form of BPDCN is less than 1% of cases of acute leukemia [2]. It affects elderly males with a male to female ratio of 3 : 1. The leukemic form presents with an aggressive behavior and rapidly disseminates without cutaneous involvement. The clinical findings include cytopenia, particularly thrombocytopenia. Although it responds to chemotherapy, the relapse is a rule and prognosis is very poor [3–6].

There is limited data published in the literature about the leukemic form, making it very problematic to define the biological and clinical features, hence, the appropriate therapeutic approach. Given the fact that there is lack of standardized therapeutic approach, we herein report a case with acute leukemic form of BPDCN who survived 48 months after autologous hematopoietic stem cell transplantation.

## 2. The Case 

A 20-year-old woman was admitted to our hospital with fatigue. The complete blood count showed pancytopenia with a hemoglobin level of 5.7 g/dL, a WBC count of 0.9 × 10^3^/*μ*L, and a platelet count of 92 × 10^3^/*μ*L. There were no significant abnormalities in the biochemical values, except for an elevated LDH level of 611 U/L. The blood coagulation tests and urine analysis were unremarkable. Peripheral blood smear was compatible with blood count. A bone marrow examination showed diffuse monotonous infiltrate of blasts and the blasts were medium size with scant agranular cytoplasm, fine chromatin, irregular nuclei, and small nucleoli. Flow cytometric analyses revealed 98.04% CD33 positivity, 96.99% HLA-DR positivity, 96.74% CD64 positivity, 97.09% CD45 positivity, 81.25% CD4 positivity, and 91.21% dual expression of CD56 and CD38 in the blastic cell population. These blasts were also negative for cCD3, CD5, CD7, CD8, CD10, CD13, CD14, CD19, CD20, CD22, CD34, cCD79a, CD103, CD117, and MPO. Conventional cytogenetic analysis showed a karyotype of 47, XX, t(7; 9)(p15; p24), +8. Diagnosis of blastic plasmacytoid dendritic cell neoplasm with leukemic form was made. Induction chemotherapy with cytarabine (3 g/m^2^/12 hours intravenous on days 1, 3, 5, and 7), idarubicin (12 mg/m^2^/day intravenous on days 1, 2, and 3), and etoposide (75 mg/m^2^/day intravenous on days 1–7) was started. On day 30 after the remission-induction treatment, analysis of bone marrow showed a normal cell lineage. Karyotype analysis was also normal. Then the patient received a consolidation with three consecutive courses of cytarabine (1.5 g/m^2^/12 hours intravenous on days 1–5). Because there was no related donor match for the patient, autologous peripheral stem cell transplantation was performed. The patient is still in complete remission after 4 years of transplantation.

## 3. Discussion

Blastic plasmocytoid dendritic cell neoplasm (BPDCN) is formerly known as CD4+/CD56+ hematodermic neoplasm. This is a rare but clinically aggressive hematologic malignancy resulting from the precursors of plasmocytoid dendritic cells. It is classified as a distinct entity under the acute myeloid leukemias in 1998 by World Health Organization [1]. Although it can be seen in pediatric population, BPDCN predominantly affects elder age groups. Since clinical presentation is indolent, it is sometimes difficult to make prompt diagnosis. Although BPDCN has a set of coherent clinical and histological findings, it frequently overlaps with other hematologic malignancies. Therefore, immunophenotyping with positive CD4+CD56+CD128+ phenotype associated with negative lineage-specific markers is required for accurate diagnosis.

There is a limited number of cases in the literature making it difficult to reach the a consensus on the optimal treatment of BPDCN. Several treatment options were tried and the majority of the patients received multiagent chemotherapy based on ALL or AML schedules. On the other hand, only a few cases underwent allogeneic HSCT [6–8]. In a study by Feuillard et al. [6], three patients underwent allogeneic HSCT after complete remission and they survived 38, 76, and 98 months, respectively. Dietrich et al. [9] reported a small case series consisting of 6 BPDCN cases. Three of the patients underwent allogeneic HSCT. They observed that relapse occurred very rapidly after a median of only 7 months if allogeneic HSCT was not performed in the first remission. In a recent study, 47 cases of BPDCN were retrospectively analysed [10]. In this cohort, they included patients with only cutaneous involvement and the only two patients who survived more than 4 years were allogeneic bone marrow transplanted. Similar to our case, autologous transplantation was performed in one patient. This patient also survived more than 38 months. In a recent Italian multicenter study, 43 patients at onset of a leukemic form of BPDCN were evaluated [11]. Allogeneic HSCT was performed in six cases. The median overall survival of these patients was 22.7 months. In addition, Ramanathan et al. [12] reported a case of BPDCN onset in a young woman as a leukemia, achieving complete remission after treatment according to regimens combining ALL-like and AML-like therapy, followed by consolidation with cord blood stem cell transplantation.

Taken together, allogeneic stem cell transplantation in the first complete remission CR should be recommended for younger patients with BPDCN. The best therapy is still unknown for the patients whose allogeneic HSCT is not possible due to limited donor availability. To the best of our knowledge this is the first case report demonstrating the successful use of peripheral autologous stem cell in this rare and aggressive disease. Our data suggest that autologous HSCT can be feasible in the absence of related donor and should be attempted in patients who are candidates for transplant.

## References

[1] Swerdlow SH, Campo E, Harris NL (2008). *WHO Classification of Tumours of Haematopoietic and Lymphoid Tissuesed*.

[2] Bueno C, Almeida J, Lucio P (2004). Incidence and characteristics of CD4(+)/HLA DRhi dendritic cell malignancies. *Haematologica*.

[3] Chen J, Zhou J, Qin D, Xu S, Yan X (2011). Blastic plasmacytoid dendritic cell neoplasm. *Journal of Clinical Oncology*.

[4] Cota C, Vale E, Viana I (2010). Cutaneous manifestations of blastic plasmacytoid dendritic cell neoplasm-morphologic and phenotypic variability in a series of 33 patients. *American Journal of Surgical Pathology*.

[5] Ferran M, Gallardo F, Ferrer AM (2008). Acute myeloid dendritic cell leukaemia with specific cutaneous involvement: a diagnostic challenge. *British Journal of Dermatology*.

[6] Feuillard J, Jacob M, Valensi F (2002). Clinical and biologic features of CD4(+)CD56(+) malignancies. *Blood*.

[7] Tsagarakis NJ, Kentrou NA, Papadimitriou KA (2010). Acute lymphoplasmacytoid dendritic cell (DC2) leukemia: results from the Hellenic Dendritic Cell Leukemia Study Group. *Leukemia Research*.

[8] Jardin F, Ruminy P, Parmentier F (2011). TET2 and TP53 mutations are frequently observed in blastic plasmacytoid dendritic cell neoplasm. *British Journal of Haematology*.

[9] Dietrich S, Andrulis M, Hegenbart U (2011). Blastic plasmacytoid dendritic cell neoplasia (BPDC) in elderly patients: results of a treatment algorithm employing allogeneic stem cell transplantation with moderately reduced conditioning intensity. *Biology of Blood and Marrow Transplantation*.

[10] Dalle S, Beylot-Barry M, Bagot M (2010). Blastic plasmacytoid dendritic cell neoplasm: is transplantation the treatment of choice?. *British Journal of Dermatology*.

[11] Pagano L, Valentini CG, Pulsoni A (2013). Blastic plasmacytoid dendritic cell neoplasm with leukemic presentation: an Italian multicenter study. *Haematologica*.

[12] Ramanathan M, Cerny J, Yu H, Woda BA, Nath R (2013). A combination treatment approach and cord blood stem cell transplant for blastic plasmacytoid dendritic cell neoplasm. *Haematologica*.

